# Mild Oxidative Damage in the Diabetic Rat Heart Is Attenuated by Glyoxalase-1 Overexpression

**DOI:** 10.3390/ijms140815724

**Published:** 2013-07-29

**Authors:** Olaf Brouwers, Joyce M. J. de Vos-Houben, Petra M. G. Niessen, Toshio Miyata, Frans van Nieuwenhoven, Ben J. A. Janssen, Geja Hageman, Coen D. A. Stehouwer, Casper G. Schalkwijk

**Affiliations:** 1 Laboratory for Metabolism and Vascular Medicine, Division of General Internal Medicine, Department of Internal Medicine, Maastricht University Medical Center, Universiteitssingel 50, PO Box 616 (#14), 6200MD Maastricht, The Netherlands; E-Mails: petra.niessen@maastrichtuniversity.nl (P.M.G.N.); cda.stehouwer@mumc.nl (C.D.A.S.); c.schalkwijk@maastrichtuniversity.nl (C.G.S.); 2 Department of Toxicology, Maastricht University Medical Center, 6200MD Maastricht, The Netherlands; E-Mails: joyce.de.vos@mumc.nl (J.M.J.V.-H.); g.hageman@maastrichtuniversity.nl (G.H.); 3 Centre of Translational and Advanced Research, Tohoku University, Sendai 980-8575, Japan; E-Mail: miyata@med.tohoku.ac.jp; 4 Department of Physiology, Maastricht University Medical Center, 6200MD Maastricht, The Netherlands; E-Mail: f.vannieuwenhoven@maastrichtuniversity.nl; 5 Department of Pharmacology, Maastricht University Medical Center, 6200MD Maastricht, The Netherlands; E-Mail: b.janssen@maastrichtuniversity.nl

**Keywords:** glycation, oxo-aldehydes, glyoxalase-I, oxidative stress, cardiac function

## Abstract

Diabetes significantly increases the risk of heart failure. The increase in advanced glycation endproducts (AGEs) and oxidative stress have been associated with diabetic cardiomyopathy. We recently demonstrated that there is a direct link between AGEs and oxidative stress. Therefore, the aim of the current study was to investigate if a reduction of AGEs by overexpression of the glycation precursor detoxifying enzyme glyoxalase-I (GLO-I) can prevent diabetes-induced oxidative damage, inflammation and fibrosis in the heart. Diabetes was induced in wild-type and GLO-I transgenic rats by streptozotocin. After 24-weeks of diabetes, cardiac function was monitored with ultrasound under isoflurane anesthesia. Blood was drawn and heart tissue was collected for further analysis. Analysis with UPLC-MSMS showed that the AGE N^ɛ^-(1-carboxymethyl)lysine and its precursor 3-deoxyglucosone were significantly elevated in the diabetic hearts. Markers of oxidative damage, inflammation, and fibrosis were mildly up-regulated in the heart of the diabetic rats and were attenuated by GLO-I overexpression. In this model of diabetes, these processes were not accompanied by significant changes in systolic heart function, *i.e.*, stroke volume, fractional shortening and ejection fraction. This study shows that 24-weeks of diabetes in rats induce early signs of mild cardiac alterations as indicated by an increase of oxidative stress, inflammation and fibrosis which are mediated, at least partially, by glycation.

## 1. Introduction

Patients with diabetes have a high risk of developing heart failure independent from traditional risk factors such as coronary artery disease and hypertension [1]. Diabetic cardiomyopathy (DCM) is characterised by diastolic dysfunction early in the disease, which can progress to compromised systolic cardiac performance and, ultimately, to cardiac failure [2]. The processes which contribute to DCM are multiple and include myocardial changes due to apoptosis, fibrosis, hypertrophy and microcirculatory abnormalities [3]. Although the precise biochemical pathways involved in DCM remain to be elucidated, it has been postulated that increased production of reactive oxygen species (ROS) by hyperglycemia is the unifying mechanism leading to diabetic cardiovascular complications [4]. Oxidative stress, defined as the imbalance between the production and the elimination of free radicals, has been shown to play a critical role in the development of heart failure and left ventricular remodeling in DCM by causing DNA damage, accelerated apoptosis, inflammation and consequently, fibrosis of the heart [5,6].

Hyperglycemia exacerbates generation of ROS. We and others have previously shown that inhibiting the formation of advanced glycation end-products (AGEs) by overexpression of the glyoxalase-1 gene can lead to decreased diabetes-induced ROS levels [7–9]. Under physiological circumstances the glyoxalase system, in which the enzyme glyoxalase-I (GLO-I) is the rate-limiting step, efficiently detoxifies the highly reactive carbonyls and AGE precursors glyoxal (GO) and methylglyoxal (MGO) to d-lactate and thereby, inhibits the formation of AGEs [10]. Increased levels of intracellular AGE precursors and AGEs are associated with oxidative stress, inflammation and fibrosis [11–14].

The aim of the current study was to investigate the effect of GLO-1 overexpression on diabetes-induced damage in the heart. We hypothesize that GLO-1 overexpression can decrease diabetes-induced glycation, oxidative stress, and inflammation in the heart and thereby, prevent fibrosis and eventually systolic cardiac dysfunction.

## 2. Results

### 2.1. Glyoxalase, Carbonyl Stress and Glycation in the Diabetic Heart

**Figure 1:**
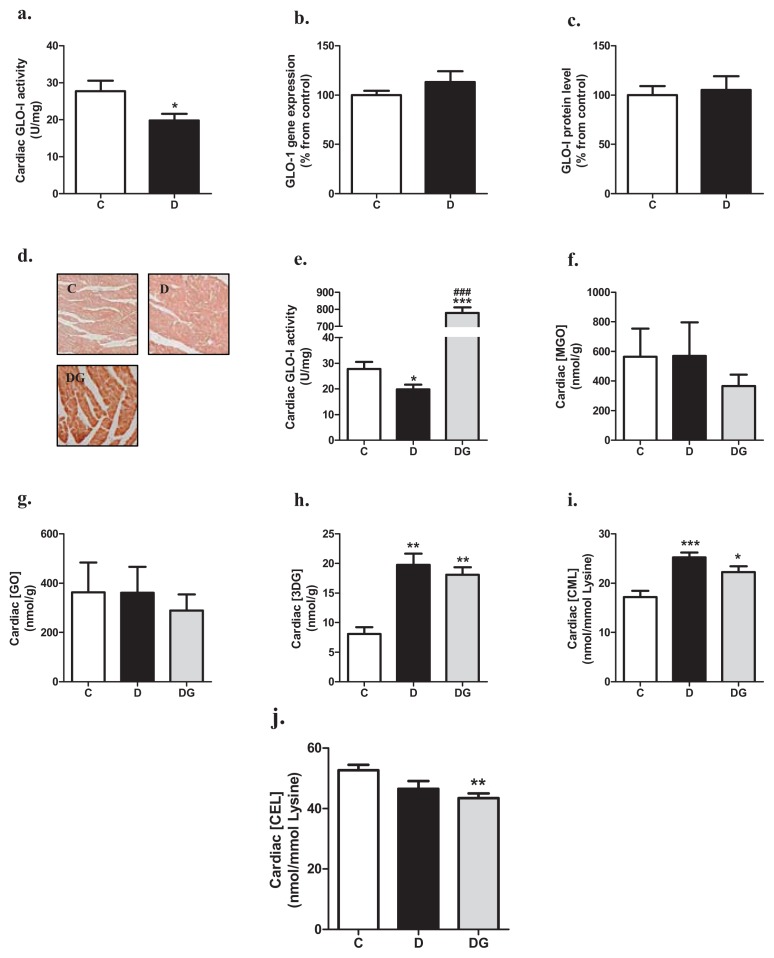
Cardiac GLO-I activity (**a**), mRNA expression (**b**) and protein levels (**c**) in control (C) and diabetic rats (D). Cardiac GLO-I overexpression was confirmed in the diabetic GLO-1 transgenic rats (DG) by making use of immunohistochemistry (**d**) and determining GLO-I enzyme activity (**e**). Cardiac levels of MGO (**f**), GO (**g**), 3-deoxyglucosone (3-DG) (**h**), N^ɛ^-(1-carboxymethyl)lysine (CML) (**i**) and N^ɛ^-(1-carboxyethyl)lysine (CEL) (**j**) in the three groups were measured by UPLC-MSMS. *p < 0.05 compared with control group, **p < 0.01 compared with control group, ***p < 0.001 compared with control group, and ^###^*p* < 0.001 compared with diabetes group.

GLO-I activity in the heart was significantly lower in diabetic rats than in control rats (Figure 1a). These changes in GLO-I activity were not accompanied by a decrease in GLO-1 mRNA expression (Figure 1b) or the active dimeric form of the GLO-I protein (Figure 1c), suggesting a post-translation effect of diabetes on GLO-I activity. In the GLO-1 overexpressing transgenic rats, GLO-I activity in the heart was approximately 25-times higher than in wild-type rats (Figure 1d,e).

After 24-weeks of hyperglycemia, levels of GO and MGO were not statistically different in the cardiac tissue of the three groups, although overexpression of GLO-1 tended to decrease levels of MGO and GO (Figure 1f,g). 3-deoxyglucosone (3-DG) was significantly increased in the diabetic heart when compared to values found in the control group, with no statistical significant effect of GLO-1 overexpression (Figure 1h). The AGE N^ɛ^-(1-carboxymethyl)lysine (CML) was significantly elevated in the heart tissue of the diabetic rats, an effect which was less pronounced in the GLO-1 transgenic group (Figure 1i). Levels of N^ɛ^-(1-carboxyethyl)lysine (CEL) tended to be decreased in the hearts of the diabetic rats compared with the control rats, which was statistically significant in the transgenic animals (Figure 1j).

### 2.2. Oxidative Damage, Inflammation and Fibrosis in the Diabetic Heart

Next, we evaluated the effect of diabetes and GLO-1 overexpression on several processes which may contribute to impaired cardiac function. Diabetes increased the levels of genes involved in oxidative stress, DNA damage, and inflammation, which was statistically significant for catalase (CAT), glutathione peroxidase-1 (GPX-1), 8-oxoguanine glycosylase (OGG-1), plasminogen activator inhibitor-1 (PAI-1), and tumor necrosis factor-α (TNFα) (Table 1). Diabetes also significantly increased the expression of connective tissue growth factor (CTGF) and decreased expression of matrix metalloproteinase-2 (MMP-2), indicating an increase of fibrosis of the heart. Furthermore, gene expression of matrix metalloproteinase-9 (MMP-9) tended to be increased and collagen type Iα (Col1α1) gene expression was significantly decreased in the diabetic heart, suggesting a compensatory mechanism to prevent fibrosis. Most of these genes were beneficially altered by GLO-1 overexpression, which was statistically significant for GPX-1, Col1α1, and MMP-2.

**Figure 2:**
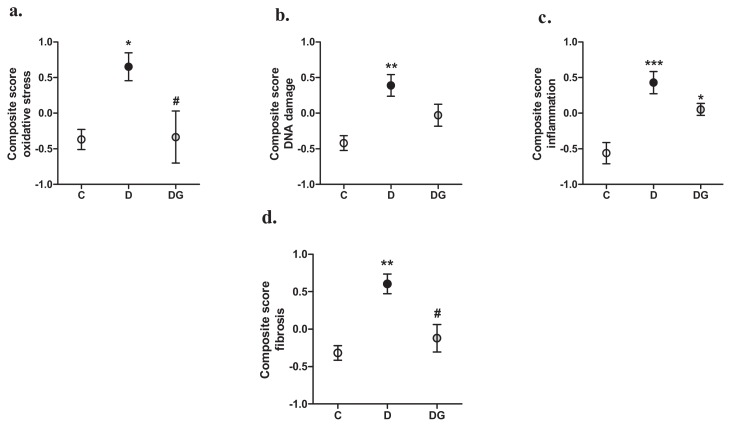
Composite scores of genes involved in oxidative stress (**a**), DNA damage (**b**), inflammation (**c**), and fibrosis (**d**) in the heart of control (C), diabetic (D) and enzyme glyoxalase-I (GLO-1) overexpressing diabetic (DG) rats. *p < 0.05 compared with control, **p < 0.01 compared with control, ***p < 0.001 compared with control, ^#^*p* < 0.05 compared with diabetes.

To obtain a good integrative measure of all these mRNA expression profiles, we calculated standardized z-scores of the individual genes and combined these in four different composite scores. These composite scores for oxidative stress, DNA damage, inflammation, and fibrosis were all significantly elevated by diabetes and decreased by GLO-1 overexpression (Figure 2). This decrease was statistically significant for the oxidative stress and fibrosis composite scores, while the composite score of DNA damage lost its statistical difference compared with the control rats. The composite score of inflammation was still significantly elevated in the transgenic diabetic rats as compared with the controls, suggesting only a partial role of glycation in this process.

**Figure 3:**
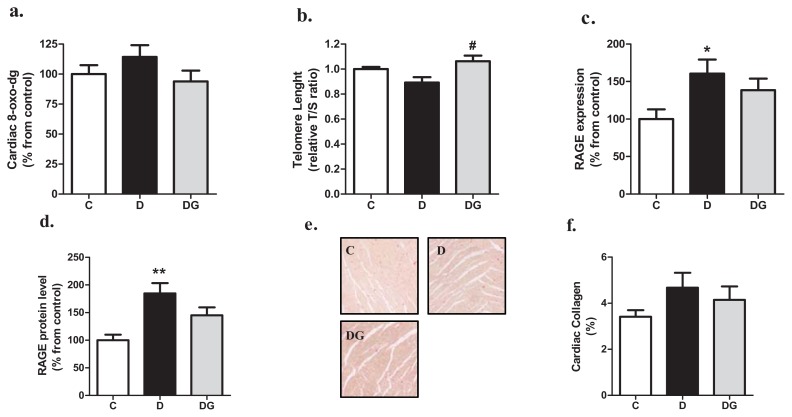
Cardiac levels of 2 markers of oxidative damage *i.e*., 8-oxo-7,8-dihydro-2′-deoxyguanosine (8-oxo-dG) (**a**), and telomere length (**b**), were measured with HPLC-ECD and real-time PCR, respectively. Key receptor for the glycation/inflammation pathway RAGE was measured on mRNA (**c**), and protein expression levels (**d**). Cardiac collagen levels in control (C), diabetic (D) and transgenic animals (DG) were visualized by Sirius red staining (**e**), and subsequently morphometrically quantified (**f**). *p < 0.05 compared with control, **p < 0.01 compared with control, ^#^*p* < 0.05 compared with diabetes.

In addition to the altered gene expression profiles we also evaluated other markers of oxidative stress, DNA damage, inflammation, and fibrosis. Hyperglycemia resulted in a tendency of mildly elevated levels of the oxidation product 8-oxo-2′-deoxyguanosine in the heart, an effect which was prevented in the GLO-1 overexpressing animals (Figure 3a). In addition, the protective effect of GLO-1 on genes involved in DNA damage is in agreement with the significant longer telomeres of the diabetic GLO-1 overexpression for rat heart compared with the wild-type diabetic rat (see Figure 3b). To address a potential diabetes-induced activation of the receptor for AGE (RAGE), we determined both mRNA and protein levels of RAGE. Gene expression of RAGE and RAGE protein increases in the diabetic heart, effects which were partially attenuated by GLO-1 overexpression (see Figure 3c,d, resp.). Finally, the diabetic heart showed a mild, but not statistically significant, increase in collagen deposition (Figure 3e) as measured by morphometric analysis of left ventricular tissue sections of the heart (Figure 3f). GLO-1 overexpression showed a tendency to decrease this marker.

### 2.3. The Diabetic Heart Displays Signs of Structural Alteration but No Systolic Dysfunction

Hyperglycemia resulted in structural alterations of the diabetic heart as indicated by an increase of organ to bodyweight ratio (LV/BW), and atrial natriuretic peptide (ANP) and brain natriuretic peptide (BNP) mRNA expression levels as compared with the control animals (Table 2). GLO-I overexpression slightly reduced this, although is not statistically significant. Nevertheless, cardiomyocytes cross-sectional surface area (CSA) did not significantly differ between the three groups (Table 2). Echocardiography revealed that the thickness and volume of the left ventricular cardiac wall were significantly decreased in both diastole as systole in the diabetic animals and this was not significantly improved by GLO-1 overexpression (Table 2). The internal diameter of the left ventricular (LV) cavity was significantly decreased during diastole in the GLO-1 overexpressing rats (Table 2). These echocardiography data reveal no LV hypertrophy, indicating that the LV/BW increase is probably caused by the strong decrease in bodyweight of the rats.

Plasma cardiac injury markers cardiac troponin I (cTnI), cardiac troponin T (cTnT), fatty acid binding protein 3 (FABP3) and myosin light chain 3 (Myl3) did not differ between the groups (data not shown). The observed plasma levels were 50 to 1000-times lower than average plasma levels of rats, which underwent the ligation of a coronary artery (data not shown), indicating that there was no relevant cardiomyocyte injury in this streptozotocin (STZ)-model.

To further characterize the diabetic heart, cardiac function of the animals was also monitored by echocardiography of the heart under isoflurane anesthesia. There were no significant differences in end diastolic volume, end systolic volume, stroke volume, fractional shortening and ejection fraction between the three groups (see Table 3). Nevertheless, diabetic animals showed a significant decrease in cardiac output, caused by a diabetes-induced bradycardia, which could not be improved by GLO-1 overexpression.

## 3. Experimental Section

### 3.1. Rat Model of Diabetes

All animal studies were carried out in accordance with the guide for the care and use of laboratory animals of the national institutes of health. All experiments involving rats were reviewed and approved by the ethics committee for animal care and use by the Maastricht University, The Netherlands.

Human GLO-1 overexpressing rats were bred as described elsewhere [7,15]. Male adult Wistar rats were divided into three groups: (i) wild type controls (C); (ii) wild type diabetics (D) and (iii) GLO-I transgenic diabetics (DG). Diabetes was induced by a single tail vein injection with 45 mg/kg streptozotocin (STZ) in saline containing 0.02 M citrate buffer (pH 4.5). After confirmation of hyperglycemia (>15 mM glucose, 5 days later) with Contour^®^ test strips (Bayer Health Care, Leverkusen, Germany), rats were included in the study and sacrificed 24-weeks after the induction of diabetes.

### 3.2. Glyoxalase-I Activity Assay

GLO-I activity was assayed by spectrophotometry (Synergy, BioTek, Winooski, VT, USA) by monitoring the increase in absorbance at 240 nm due to the formation of *S*-D-lactoylglutathione for 10 min at 37 °C according to the method of McLellan and Thornalley [16].

### 3.3. Measurement of AGEs and Oxo-Aldehydes in the Heart

The AGEs, CML and (CEL), were measured with ultra-performance liquid chromatography tandem mass spectrometry (UPLC-MSMS) as described earlier [17]. Oxo-aldehydes GO, MGO and 3-DG were assayed by derivatization with o-phenyl-enediamine (o-PD) and UPLC-MSMS as described earlier [18]. Liquid chromatography was performed at 30 °C using an Acquity UPLC BEH C18, 1.7 μm, 2.1 × 100 mm column (Waters, Milford, MA, USA), and the Micromass Quattro Premier XE Tandem Mass Spectrometer (Waters) was used in electrospray positive multiple reaction monitoring mode.

### 3.4. Cardiac Gene Expression Analysis

Total cellular RNA was extracted from ventricular heart lysates using TRIzol^®^ isolation (Invitrogen, Life Technologies Ltd., Paisley, UK). The amounts of RNA extracted were quantified by measuring the absorbance at 260 nm by spectrophotometry (NanoDrop^®^, Thermo Scientific, Wilmington, NC, USA). Reverse transcription from RNA to DNA was performed with a Reverse Transcriptase kit from Bio-Rad (Hercules, CA, USA) under the following conditions: 25 °C for 10 min, 48 °C for 30 min and 94 °C for 30 s. The PCR was performed in a volume of 25 μL in each well containing RNA, Quanta Universal PCR MasterMix (Quanta BioSciences, Gaithersburg, MA, USA) and primers of the target of interest, all purchased from Eurogentec (Liège, Belgium). The following genes were evaluated; superoxide dismutase-3 (SOD-3), CAT, GPX-1, nicotinamide adenine dinucleotide phosphate-oxidase-4 (NOX-4), E1A binding protein p300 (p300), DNA-(apurinic)lyase (APEX-1), OGG-1, tumor protein 53 (p53), PAI-1, TNFα, interleukin-1β (IL-1β), interleukin-6 (IL-6), CTGF, MMP-2, MMP-9, Col1α1, ANP, and BNP. β-actin was used as reference gene (Table 4).

Each real time PCR reaction ran at 50 °C for 2 min, 95 °C for 10 min and in 40 cycles changing between 95 °C for 15 s and 60 °C for 90 s. Data were analyzed with the ABI Prism 7000 Sequence Detector Software (Applied Biosystems, Life Technologies Ltd., Paisley, UK). The output of amplification was measured in the exponential phase of the reaction as the threshold cycle/Ct-value, which is defined as the cycle number at which amplification products are detected, corresponding to the point where fluorescent intensity exceeds the background fluorescent intensity, which is 10-times the standard deviation of the baseline. The relative quantification of target gene was calculated using the formula: (1/2)Ct-target gene- Ct-housekeeping gene.

### 3.5. DNA Isolation and Telomere Length Measurement

DNA was extracted using the QIAamp DNA Mini Kit (Qiagen, Venlo, The Netherlands) according to the manufacturer’s protocol and quantified using a spectrophotometry (NanoDrop^®^, Thermo Scientific, Wilmington, NC, USA). Telomere length (TL) was determined by quantitative PCR as described by Cawthon [19]. Two master mixtures were prepared, one with telomere primers and one with phosphoglycerate kinase 1 (PGK1) primers (1× IQ SYBRgreen supermix from Bio-Rad, Hercules, CA, USA). Sequences of the primers are shown in Table 4. The relative TL was calculated as the ratio of telomere repeats to single-copy gene copies (*T*/*S* ratio). This means that *T*/*S* = 1 when the unknown DNA is identical to the reference DNA in its ratio of telomere repeat copy number to single copy gene copy number [20].

### 3.6. Western Blotting Analyses

Total protein was isolated by homogenizing 50 mg ventricular tissue in 1mL sodium phosphate buffer with 0.02% Triton (pH 7.0) and protease inhibitors (Mini Cocktail, Roche Diagnostics, Indianapolis, IN, USA). Protein concentration was determined using a BCA kit (Pierce, Rockford, IL, USA). After boiling in loading buffer, 15 μg of protein was loaded onto a 12% SDS-polyacrylamide gel electrophoresis (SDS-PAGE), and transferred to a PVDF membrane. Blots were blocked with 5% nonfat dry milk containing phosphate buffered saline (for RAGE) or 5% BSA Cohn fraction V containing Tris-buffered saline solution (for GLO-I) supplemented with 0.1% Tween-20. Incubation with one of the following primary antibodies was performed at room temperature for 1 h: rabbit-anti–GLO-I (1:2000); rabbit-anti-RAGE (1:2000); or mouse-anti-total actin (1:10000). Incubation with either a secondary anti-rabbit-, or anti-mouse-conjugated horseradish peroxidase antibody (DAKO, Glostrup, Denmark)) was also performed at room temperature for 1 h. After washing in buffered saline and 0.1% Tween-20, blots were exposed to ECL Plus (Pierce, ThermoScientific, Rockford, IL, USA) and bands were quantified by using the Chemidoc system and the software program Quantity One (Biorad) Both GLO-I and RAGE intensities were normalized for total actin.

### 3.7. 8-Oxo-dG Quantification in the Heart

The base-oxidation product 8-oxo-7,8-dihydro-2′-deoxyguanosine (8-oxo-dG) was detected using HPLC with electrochemical detection (ECD). Measurements were performed as described earlier [21]. Genomic DNA was obtained by grinding approx. 50mg frozen tissue followed by phenol extraction. The DNA-extraction procedure was optimized to minimize artificial induction of 8-oxo-dG, by using radical-free phenol, minimizing exposure to oxygen and by addition of 1 mM deferoxamine mesylate and 20 mM TEMPO (2,2,6,6-tetramethylpiperidine-*N*-oxyl). HPLC-ECD of 8-oxo-dG was performed by digesting 30 μg DNA to deoxyribonucleosides by addition of 6 μL 0.5 M NaAc, 9 μL 10 mM ZnCl_2_ and 1.5 μL nuclease P1 (stock: 1 U/μL) and incubation for 90 min at 37 °C. Subsequently, 30 μL 0.5 M Tris-HCl (pH 7.4) and 1.5 μL alkaline phosphatase (0.014 U/μL) were added followed by incubation at 37 °C for 45 min. The digest was then analyzed by HPLC-ECD on a Supelcosil™ LC-18S column (250 mm × 4.6 mm) (Supelco Park, Bellefonte, PA, USA) in combination with a DECADE electrochemical detector (Antec, Leiden, The Netherlands). The ECD-signal was first stabilized with mobile phase (94 mM KH_2_PO_4_, 13 mM K_2_HPO_4_, 26 mM KCl and 0.5 mM EDTA, 10% methanol) for approximately 3 h at a flow rate of 1 mL/min. After stabilization, 8-oxo-dG was detected at a potential of 400 mV and dG was simultaneously monitored by UV absorption at 260 nm.

### 3.8. Heart Tissue Analyses for Cardiomyocyte Area and Collagen

For immunohistochemistry, hearts were fixed with phosphate-buffered (pH 7.4) formaldehyde (4%) overnight at room temperature. Subsequently, hearts were transferred to 70% ethanol embedded in paraffin and processed for histological examination. For this, tissue sections of 5 μm were fixed at 56 °C overnight, deparaffinised, rehydrated and stained with haematoxylin and eosin (H&E, Sigma Aldrich, St. Louis, MO, USA) to determine cardiomyocyte cross-sectional surface area using Qwin version 3 morphometric software [22] (Leica, Cambridge, UK). To visualize interstitial fibrosis, the sections were stained with Picro-Sirius Red. The percentage of the LV wall consisting of interstitial collagen was calculated as the ratio of Picro-Sirius-Red positively stained area over total LV tissue area, excluding blood vessels, using Qwin software (Leica, Cambridge, UK).

### 3.9. Measurement of Circulating Cardiac Injury Markers

Rat cardiac injury panel 3 was purchased from Meso Scale Discovery (Meso Scale Discovery, Rockville, MD, USA). This array detects cTnT, cTnI, Myl3 and FABP3 in a sandwich immunoassay. Each 96-well plate had 4-carbon electrodes in the bottom of each well; each pre-coated with one of the 4-anti-cardiac injury markers antibodies of interest.

### 3.10. Echocardiographic Analyses of Cardiac Dimensions and Function

LV dimensions and function were assessed under isoflurane anesthesia (2%–3% isoflurane) as described earlier [23]. Shortly B-mode echocardiographic recordings were made in midpapillary short-axis and parasternal long-axis using a Vevo2100 imaging platform (Visual Sonics, Toronto, Canada). Data were derived from images in end diastole and peak systole and average values over at least three different cycles were used. From heart rate and differences in internal LV diameter during systole and diastole, stroke volume and cardiac output were calculated, assuming that the ventricular cavity is ellipsoid in shape.

### 3.11. Statistical Analysis

All values are expressed as mean ± SEM. The statistical differences between groups were tested using one-way ANOVA with a post-hoc Bonferroni correction for the groups of interest, or students *t*-test when appropriate. A *p*-value of less than 0.05 was considered statistically significant. We also calculated a composite score of oxidative stress markers (SOD-3, CAT, GPX-1 and NOX-4), DNA damage markers (p300, APEX-1, OGG-1 and p53), inflammation markers (PAI-1, TNFα, IL-1β and IL-6), and fibrosis markers (CTGF, Col1α1, MMP-2 and MMP-9) by averaging the z-scores of each of the respective markers to an overall composite score. Each z-score represents the distance between the raw score from the total mean in units of the standard deviation. The obtained composite scores thus represent a good integral measure of the processes/mechanisms and have the advantage of reducing the influence of the biological variability when each of their constituent markers is tested separately.

## 4. Discussion and Conclusions

In this model of diabetes we found that 24-weeks of diabetes had mild effects on the processes of oxidative damage, inflammation and fibrosis in the heart. The systolic function of the heart was not altered. Overexpression of GLO-1 partially prevented this mild increase in oxidative damage, inflammation and fibrosis, but did not have clear effects on the cardiac dimensions.

We previously reported that STZ-induced diabetes in rats leads to an increase of dicarbonyls and glycation in the plasma, blood vessels and retina, and that this could be partially prevented by GLO-I overexpression [7,15]. In the present study we show that levels of CML, and not CEL, are elevated in the heart of the diabetic rats. CML is an AGE which can be derived from both lipid oxidation and glycolysis, while the AGE CEL is predominantly glycolysis-derived. Because diabetes mellitus is characterized by reduced glucose metabolism and enhanced fatty acid (FA) metabolism, it is likely that FA uptake exceeds oxidation rates in the heart, thereby resulting in lipid accumulation in the myocardium that may promote lipotoxicity, and thereby, a production of the lipid oxidation product CML. Thus, the shift in myocardial substrate and energy metabolism may contribute to increased levels of CML and decreased levels of CEL. The diabetic heart is also characterized by increased levels of fructose, leading to the accumulation of fructose-3-phosphate and its decomposition product 3-DG [24,25]. In accordance, also in our study, we found increased levels of 3DG in the diabetic heart.

Diabetes is associated with increased oxidative (DNA) damage and impaired DNA repair leading to an accelerated stage of aging [26]. Several studies showed that FA and glycation intermediates can lead to oxidative damage of vascular cells and especially MGO can be designated as an intermediate which potentially leads to damage of the DNA [27]. In our study, the antioxidant genes catalase (CAT) and glutathione peroxidase-1 (GPX-1) were elevated by diabetes, indicating an activated defense mechanism against superoxides and hydrogen peroxides. Also, the expression of genes involved in DNA repair *i.e.*, p300, APEX-1, OGG-1 and p53, showed a tendency to be elevated by diabetes, suggesting a mild diabetes-induced increase in oxidative DNA damage. Indeed, also two markers of DNA damage, *i.e.*, increased 8-oxo-2-dG and decreased telomere length (TL), were mildly altered in the STZ rats, and overexpression of GLO-1 overall prevented this. Cardiac DNA damage potentially plays a role in diabetes-induced cardiac dysfunction since people with diabetes have shorter TL, and this is associated with left ventricular dysfunction [28,29]. Our data thus suggest that diabetes-induced cardiac DNA damage is partly regulated by glycation.

In the diabetic heart the pro-oxidant and pro-inflammatory environment can lead to fibrosis, and consequent stiffening of the cardiac muscle. Indeed, not only the gene-expression of TNFα, IL-1β and IL-6, but also the expression of CTGF, and mild signs of fibrosis were increased in the heart by diabetes. This effect might be due to the increase in CML levels and activation of RAGE dependent inflammation pathways [14]. The effect of GLO-1 overexpression on the expression pattern of RAGE suggests that the beneficial effects of GLO-I are at least partially mediated by inhibiting the CML/RAGE pathway. Despite the similar finding of Yao *et al.*, that GLO-1 overexpression prevents high glucose-induced RAGE elevation *in vitro* [30], this crosstalk between the GLO-I and RAGE pathways deserves further investigation.

The collagen area in left ventricular myocardial tissue of the STZ rats was increased by ~40%, which is comparable with earlier findings of other groups [31,32]. The decreased expression of Col1α1, suggest that alterations in the rate of collagen degradation rather than enhanced synthesis are responsible for the mild degree of fibrosis. Collagen degradation is largely regulated by MMPs. Both MMP-2 and MMP-9 are matrix proteases known to be involved in myocardial remodeling. The increased gene expression level of MMP-9 would favor matrix degradation, while the decreased MMP-2 expression would favor the development of fibrosis. Similar decreased expression of Col1α1, and MMP-2, and increased MMP-9 expression have been observed before in diabetic rodents, and were associated with cardiac fibrosis [33–35].

Our current study shows that, despite mild alterations in gene expression and morphology of the heart, 24 weeks of diabetes did not affect cardiac systolic function, regarding the amount of stroke volume produced by the heart. The fractional shortening and ejection fraction were also not altered by the STZ-injection. However, we and others already showed earlier that heart rate of the diabetic rats is decreased, which in this study resulted in a decreased calculated cardiac output, without any effect of the GLO-I overexpression [36]. Data about the effect of STZ on cardiac function and especially diabetes-induced left ventricular dysfunction are conflicting. Some studies report early systolic dysfunction of the left ventricular in STZ-induced diabetic cardiomyopathy [37,38], while other studies [39,40], including our own data, showed no differences in LV function between diabetic and control rats. A study of Hoit *et al.* did not see any basal contractile dysfunction in STZ rats, only after isoproterenol stimulation, indicating a masked cardiac dysfunction [41]. Differences in strain, timeframe, or detection method can also contribute to the divergent observations [42]. Also, alterations in cardiac diastolic function are not unambiguously found in other animal models of diabetes [35,43].

In conclusion, this study shows that 24 weeks of diabetes induces mild cardiac alterations, which is partially caused by glycation, however, without significantly effecting cardiac function.

## Figures and Tables

**Table 1:** mRNA expression levels of genes involved in oxidative stress, DNA damage, inflammation and fibrosis. Values are given as percentage from control.

Genes	Control	Diabetes	Diabetes + GLO-I
Oxidative stress	(C)	(D)	(DG)
SOD-3	100 ± 9	122 ± 9	91 ± 14
CAT	100 ± 10	226 ± 46 *	190 ± 43
GPX-1	100 ± 6	149 ± 12 *	91 ± 12 #
NOX-4	100 ± 9	130 ± 9	97 ± 17
DNA damage	-	-	-
p300	100 ± 6	116 ± 6	94 ± 16
APEX-1	100 ± 1	121 ± 9	104 ± 8
OGG-1	100 ± 6	121 ± 4 *	129 ± 13
p53	100 ± 25	133 ± 29	133 ± 33
Inflammation	-	-	-
PAI-1	100 ± 13	278 ± 28 ***	359 ± 46 ***
TNFα	100 ± 12	126 ± 17	114 ± 17
IL-1β	100 ± 11	216 ± 26 *	162 ± 28
IL-6	100 ± 23	108 ± 19	93 ± 18
Fibrosis	-	-	-
CTGF	100 ± 13	180 ± 18 ***	197 ± 23
Col1α1	100 ± 13	65 ± 7 *	109 ± 14 #
MMP-2	100 ± 6	78 ± 6 *	115 ± 10 #
MMP-9	100 ± 23	150 ± 35	128 ± 22

* *p* < 0.05 compared with control,

** *p* < 0.01 compared with control,

*** *p* < 0.001 compared with control,

# *p* < 0.05 compared with diabetes.

**Table 2:** The effect of diabetes and GLO-1 overexpression on several parameters of cardiac remodeling. Gene expression values are given as percentage from control.

Characteristic	Control	Diabetes	Diabetes + GLO-I
Cardiac Hypertrophy	(C)	(D)	(DG)
Cardiac weight (g)	1.32 ± 0.08	1.080 ± 0.05 *	0.993 ± 0.04 **
Heart/bodyweight (%)	0.276 ± 0.01	0.360 ± 0.01 ***	0.333 ± 0.01 **
ANP (% from C)	100 ± 15	499 ± 139 **	385 ± 48 ***
BNP (% from C)	100 ± 16	148 ± 23 *	157 ± 13 **
Cardiomyocyte CSA (μm^3^)	277 ± 12	284 ± 21	260 ± 21
Ventricular Dimensions	-	-	-
Thickness wall diastole (cm)	0.17 ± 0.01	0.14 ± 0.01 **	0.15 ± 0.01 *
Volume wall diastole (cm^3^)	0.49 ± 0.03	0.40 ± 0.03 *	0.45 ± 0.02
Internal diameter cavity diastole (cm)	0.84 ± 0.06	0.84 ± 0.05	0.77 ± 0.04 **
Thickness wall systole (cm)	0.19 ± 0.01	0.17 ± 0.01 **	0.17 ± 0.01 *
Volume wall systole (cm^3^)	0.43 ± 0.03	0.39 ± 0.02	0.40 ± 0.02
Internal diameter cavity systole (cm)	0.68 ± 0.06	0.70 ± 0.04	0.64 ± 0.04

* *p* < 0.05 compared with control,

** *p* < 0.01 compared with control,

*** *p* < 0.001 compared with control.

**Table 3:** The effect of diabetes and GLO-1 overexpression on cardiac volume and function.

Characteristic	Control	Diabetes	Diabetes + GLO-I
Cardiac volume	(C)	(D)	(DG)
End diastolic volume (mL)	0.534 ± 0.04	0.536 ± 0.04	0.492 ± 0.02
End systolic volume (mL)	0.211 ± 0.02	0.218 ± 0.01	0.205 ± 0.01
Stroke volume (mL)	0.322 ± 0.03	0.318 ± 0.03	0.287 ± 0.02
Cardiac function	-	-	-
Fractional shortening (%)	16.8 ± 1.0	16.0 ± 1.2	16.6 ± 0.8
Ejection fraction (%)	59.4 ± 3.1	58.9 ± 1.3	58.0 ± 2.2
Cardiac output (mL/min)	110.7 ± 9.7	81.2 ± 7.7	77.8 ± 6.5 *

* *p* < 0.05 compared with control.

**Table 4:** Sequences of primers used.

Genes	Forward primer	Reverse primer
Oxidative stress	**-**	**-**
SOD-3	CTTCCCAGCCGAGCAGAA	TCAGGTCCCCGAACTCATG
CAT	TCCTGAGAGAGTGGTACATGC	AAGGTGTGAGCCATAGCC
GPX-1	AATTCCCTCAAGTACGTCCG	TCAGGTTCGATGTCGATGGT
NOX-4	TGGGTGTCAAACAAGAGATTGC	GGCTCAGGAGGTTCTTCATGTAG
DNA Damage	-	-
p300	GGGACTAACCAATGGTGGTG	ATTGGGAGAAGTCAAGCCTG
APEX-1	GAATGTGGATGGGCTTCGA	AAGATGTCTGGTGCTTCTTCCTTT
OGG-1	CGGCTGGCAGCCTAAGAC	CCGGAAAAAGTTTCCCAGTTC
p53	GACAGCTTTGAGGTTCGTGTTTG	TGCTCTTCTTTTTTGCGGAAA
Inflammation	-	-
PAI-1	TCCGCCATCACCAACATTTT	GTCAGTCATGCCCAGCTTCTC
TNFα	TGATCGGTCCCAACAAGGA	GGGCCATGGAACTGATGAGA
IL-1β	GCACCTTCTTTTCCTTCATCTTTG	CACACTAGCAGGTCGTCATCATC
IL-6	GAAACCCTAGTTCATATCTTCAAACAAG	CCATTAGGAGAGCATTGGAAGTTG
Fibrosis	-	-
CTGF	CACAGAGTGGAGCGCCTGTTC	GATGCACTTTTTGCCCTTCTTAATG
Col1α1	CGAAGGCAACAGTCGATTCA	GGTCTTGGTGGTTTTGTATTCGAT
MMP-2	TTTGCTCGGGCCTTAAAAGTAT	CCATCAAACGGGTATCCATCTC
MMP-9	GACCTGAAAACCTCCAACCTC	CTGCTTCTCTCCCATCATCTG
Hypertrophy	-	-
ANP	ATCACCAAGGGCTTCTTCCT	TGTTGGACACCGCACTGTAT
BNP	AGACAGCTCTCAAAGGACCA	CTATCTTCTGCCCAAAGCAG
Telomere length	-	-
PGK1	CGGAGACACCFCCACTTG	AAGGCAGGAAAATACTAAACAT
Telomere 1	CGGTTTGTTTGGGTTTGGGTTTGGGTTTGGGTTTGGGTT	
Telomere 2	GGCTTGCCTTACCCTTACCCTTACCCTTACCCTTACCCT	
Reference gene	*-*	*-*
β-actin	GACAGGATGCAGAAGGAGATTACTG	CCACCGATCCACACAGAGTACTT
